# Routine HIV Screening in France: Clinical Impact and Cost-Effectiveness

**DOI:** 10.1371/journal.pone.0013132

**Published:** 2010-10-01

**Authors:** Yazdan Yazdanpanah, Caroline E. Sloan, Cécile Charlois-Ou, Stéphane Le Vu, Caroline Semaille, Dominique Costagliola, Josiane Pillonel, Anne-Isabelle Poullié, Olivier Scemama, Sylvie Deuffic-Burban, Elena Losina, Rochelle P. Walensky, Kenneth A. Freedberg, A. David Paltiel

**Affiliations:** 1 Service Universitaire des Maladies Infectieuses et du Voyageur, Centre Hospitalier de Tourcoing, Tourcoing, France; 2 Avenir- Action Thématique et Incitative sur Programme, Institut National de la Santé et de la Recherche Médicale U995, Lille, France; 3 EA 2694, Faculté de Médecine de Lille, Lille, France; 4 Division of General Medicine, Massachusetts General Hospital, Boston, Massachusetts, United States of America; 5 Division of Infectious Diseases, Massachusetts General Hospital, Boston, Massachusetts, United States of America; 6 CRESGE-LEM, Centre National de la Recherche Scientifique UMR8179, Lille, France; 7 HIV/AIDS-STI-HCV Unit, Department of Infectious Diseases, Institut de Veille Sanitaire, St. Maurice, France; 8 Institut National de la Santé et de la Recherche Médicale U943, Paris, France; 9 Institut National de la Santé et de la Recherche Médicale UMR S 943, Paris, France; 10 Université Paris 06 Pierre et Marie Curie, Paris, France; 11 Service des Maladies Infectieuses et Tropicales, Assistance Publique – Hôpitaux de Paris, Groupe hospitalier Pitié-Salpétrière, Paris, France; 12 Economic and Public Health Assessment Department, Haute Autorité de Santé, Saint Denis, France; 13 Institut National de la Santé et de la Recherche Médicale U995, Faculté de Médecine de Lille, Lille, France; 14 Departments of Biostatistics and Epidemiology, Boston University School of Public Health, Boston, Massachusetts, United States of America; 15 Department of Orthopedic Surgery, Brigham and Women's Hospital, Boston, Massachusetts, United States of America; 16 Division of Infectious Diseases, Brigham and Women's Hospital, Boston, Massachusetts, United States of America; 17 Center for AIDS Research, Harvard Medical School, Boston, Massachusetts, United States of America; 18 Yale University School of Medicine, New Haven, Connecticut, United States of America; Universidade Federal de Minas Gerais, Brazil

## Abstract

**Background:**

In France, roughly 40,000 HIV-infected persons are unaware of their HIV infection. Although previous studies have evaluated the cost-effectiveness of routine HIV screening in the United States, differences in both the epidemiology of infection and HIV testing behaviors warrant a setting-specific analysis for France.

**Methods/Principal Findings:**

We estimated the life expectancy (LE), cost and cost-effectiveness of alternative HIV screening strategies in the French general population and high-risk sub-populations using a computer model of HIV detection and treatment, coupled with French national clinical and economic data. We compared risk-factor-based HIV testing (“current practice”) to universal routine, voluntary HIV screening in adults aged 18–69. Screening frequencies ranged from once to annually. Input data included mean age (42 years), undiagnosed HIV prevalence (0.10%), annual HIV incidence (0.01%), test acceptance (79%), linkage to care (75%) and cost/test (€43). We performed sensitivity analyses on HIV prevalence and incidence, cost estimates, and the transmission benefits of ART. “Current practice” produced LEs of 242.82 quality-adjusted life months (QALM) among HIV-infected persons and 268.77 QALM in the general population. Adding a one-time HIV screen increased LE by 0.01 QALM in the general population and increased costs by €50/person, for a cost-effectiveness ratio (CER) of €57,400 per quality-adjusted life year (QALY). More frequent screening in the general population increased survival, costs and CERs. Among injection drug users (prevalence 6.17%; incidence 0.17%/year) and in French Guyana (prevalence 0.41%; incidence 0.35%/year), annual screening compared to every five years produced CERs of €51,200 and €46,500/QALY.

**Conclusions/Significance:**

One-time routine HIV screening in France improves survival compared to “current practice” and compares favorably to other screening interventions recommended in Western Europe. In higher-risk groups, more frequent screening is economically justifiable.

## Introduction

An estimated 6,500 to 7,600 new cases of HIV were diagnosed every year between 2003 and 2008 in France, where the overall population size is 63 million. In 2008, 60% of those new diagnoses were among heterosexual men and women [1]. Although HIV tests are free of charge in France and current HIV testing rates among non-blood donors rank second in Europe at 5 million tests per year [2], [3], roughly 40,000 of an estimated 106,000–134,000 HIV-infected people throughout the country remain unaware of their infection [4]. Furthermore, 36% of HIV-infected patients in France present to care with CD4 counts <200/µl and/or AIDS-related symptoms [5].

Most European countries currently recommend risk-factor-based testing, wherein physicians offer HIV tests at the patient's request or when s/he is observed to be at high risk of infection [6]. Several recent studies in the United States, however, have shown that routine, voluntary HIV screening is clinically effective and cost-effective compared to *ad hoc* practices of HIV testing [7], [8]. Although challenges to implementation remain [9], the US Department of Health and Human Services recommends routine screening countrywide [10]. Given the lower prevalence of undiagnosed HIV and higher rates of non-routine HIV testing in France, however, it is not possible to extrapolate results from the United States to France.

Recognizing the need to reconsider the approach to HIV testing in France, we estimate the survival benefits, costs and cost-effectiveness of routine, voluntary HIV screening in the French general population and important sub-populations.

## Methods

### Analytic overview

We used a computer-based simulation model of HIV detection and treatment [7], [8], [11], [12] to estimate the changes in life expectancy, quality-adjusted life expectancy, and cost associated with a population-wide program of routine HIV screening once, every five years, and annually in adults aged 18–69. We also considered targeted screening in three sub-populations (men who have sex with men [MSM], injection drug users [IDU] and heterosexuals), as well as French Guyana, the French administrative region with the highest rates of HIV prevalence and delayed access to care [5], [13]. Model input parameters were derived primarily from French national data and the medical literature. Outcome measures were assessed from a modified societal perspective and life expectancy, quality-adjusted life expectancy, and costs (2007 €) were discounted at a rate of 3% per annum [14].

### Model overview

#### Disease Module

The Cost-Effectiveness of Preventing AIDS Complications (CEPAC) model is a widely published first-order state-transition Monte Carlo simulation of the natural history, clinical management, outcomes, and costs of HIV disease (see Appendix S1) [11], [12], [15]. Each HIV-infected patient is followed from model entry until death. Monthly transitions between “health states” describe the natural history of disease. Disease progression is determined by CD4 count, HIV RNA level, and history of opportunistic diseases (Appendix S1, Table A1). ART can alter these outcomes by reducing HIV RNA, increasing CD4 counts, and providing independent protection from opportunistic diseases [16]. ART and opportunistic disease prophylaxis can also lead to adverse events, resulting in increased costs and morbidity. Morbidity is incorporated in a single outcome measure which adjusts survival for quality of life [11], [12], [17], [18].

#### Screening Module

The Screening Module captures HIV prevalence and incidence and determines entry into the Disease Module (see Appendix S1) [7], [8]. HIV-infected patients can be diagnosed in three ways. First, patients can present to care with an AIDS-defining opportunistic disease. Second, they can be diagnosed via existing programs of risk-factor-based, non-routine HIV testing, hereinafter referred to as “current practice.” In the “current practice” scenario, we assume a constant rate of HIV diagnosis over both time and any expanded HIV screening intervention. Third, they can be diagnosed via an expanded program of routine screening. We assume that test sensitivity and specificity, follow-up, and linkage to care are all imperfect in the routine screening program.

The Screening Module conveys information on each patient to the Disease Module, which determines when patients who have been diagnosed and linked to care become eligible for clinic visits, ART and opportunistic disease prophylaxis [19]. Patients only initiate care once their HIV infection has been diagnosed. The delay from HIV infection to diagnosis affects the severity of disease (CD4 count) at treatment initiation.

### Disease Module inputs

#### Cohort characteristics and disease progression

The demographics of the simulated cohort represent the 18–69 year-old population in France [20]. Mean age was 42 years and 50% of participants were male (Table 1) [20]. CD4 count-stratified opportunistic disease incidence and mortality rates were derived from two French clinical cohorts [21].

**Table 1:** Summary of input parameters for a model of routine, voluntary HIV screening in France.

Variable		Baseline value	Range	Source
Age, years		42	20 – 42	[20]
Male sex, % of patients		50	---	[20]
**Prevalence of undiagnosed HIV, %**				
	General population	0.10	0.05 – 5.0	[29]–[34], [42], [62]
	Injection drug users	6.17	6.17 – 9.25	[30], [42], [62]
	French Guyana a	0.41	---	[13]
	Men who have sex with men	1.70	0.85 – 1.70	[30], [42], [62]
	Heterosexual population	0.04	---	[30], [42], [62]
**Annual incidence, /100PY**				
	General population	0.01	0.01– 0.13	[31], [34]
	Injection drug users	0.17	---	[31], [34]
	French Guyana	0.35	---	[13], [31]
	Men who have sex with men	0.99	---	[31], [34]
	Heterosexual population	0.01	---	[31], [34]
**Mean CD4 count at HIV care initiation in the “current practice” scenario, cells/**µ**l (SD)**				
	General population	372 (257)	---	[42]
	Injection drug users	342 (180)	---	[42]
	French Guyana	347 (229)	---	[42]
	Men who have sex with men	442 (289)	---	[42]
	Heterosexual population	357 (252)	---	[42]
**Monthly probability of diagnosis and linkage to care via non-routine HIV test, %**		2.8	0 – 8.3	[42]
**Rate of test acceptance, %**		79	20 – 90	[36]
**Rate of return for results and linkage to care, %**		75	20 – 90	[37]
**Costs, 2007 €**				
	Test (pre-test counseling + blood draw + ELISA)	43	11 – 85	[38]
	Confirmatory test (blood draw + Western Blot)	53	---	[38]
	Post-test linkage and counseling costs for HIV+ patients	22	---	[63]
**Secondary HIV transmission rate according to plasma viral load (copies/ml), /100PY**				
	≥50,000	9.0	4.5 – 18.1	[40]
	10,000 – 49,999	8.1	4.1 – 16.2	[40]
	3,500 – 9,999	4.2	2.1 – 8.3	[40]
	400 – 3,499	2.1	1.0 – 4.1	[40]
	<400	0.2	0.1 – 0.3	[40]
**ART efficacy at 48 weeks, % HIV RNA <400 copies/ml (mean increase in CD4 count, cells/**µ**l)**				
	TDF/FTC + EFV	81 (190)	---	[22]
	ATV/r + 2 NRTIs	70 (110)	---	[23]
	3rd-line b	58 (121 )	60 – 90	[23]
	4th-line b	65^c^ (102^c^)	50 – 70	[24]
	5th-line b	40^c^ (121)	20 – 50	[25], [47]
	6th-line b	12 (45)	10 – 40	[25]

PY: person-year; SD: standard deviation; ART: antiretroviral therapy; TDF: tenofovir; FTC: emtricitabine; EFV: efavirenz; ATV/r: ritonavir-boosted atazanavir; NRTI: nucleoside reverse transcriptase inhibitor.

a The method used to derive the prevalence of HIV in French Guyana is different than the method used for the French general population and all other sub-populations.

b Once patients start third-line therapy, genotype tests generally determine individualized regimens. ART lines 3–6 are therefore modeled as generic regimens with wide ranges of efficacy, represented by various recent studies.

c at 24 weeks.

#### Treatment

As recommended by French national guidelines, detected patients initiated ART at CD4 counts <350/µl or an observed severe opportunistic disease [19]. Patients received up to six sequential ART regimens. These were switched upon virologic failure, defined as an observed increase in detectable HIV RNA over two consecutive months. Clinic visits, CD4 counts, and HIV RNA tests occurred every three months, as well as in the month of any opportunistic disease [19]. ART and opportunistic disease prophylaxis efficacies were derived from published randomized controlled trials (Table 1) [22]–[25].

#### Costs and quality of life

The direct costs of routine medical care and opportunistic disease treatment were derived from the French Tourcoing AIDS Reference clinical cohort [26]. Although the economic cost data reported in our paper are the result of a unit costing analysis conducted on 2005 data, French health care prices have remained relatively stable in the intervening period. We used the “Health” component of the French Consumer Price Index to convert these costs to 2007. Prophylaxis and antiretroviral medication costs are from the pharmacy records of the Tourcoing Hospital in France. Quality of life weights by health state are from the HIV Cost and Services Utilization Study and other published studies (Appendix S1, Table A1) [27], [28].

### Screening Module inputs

#### Prevalence and incidence

We used two methods to estimate the prevalence of undiagnosed HIV in France in 2005. First, we employed a “back-calculation” approach – using published estimates of observed AIDS cases, the HIV-to-AIDS incubation time, and ART efficacy [29] – to estimate the number of HIV-infected persons in France in 2000 at 88,200 [29], [30]. Second, we extrapolated from non-correlated epidemiological estimates of HIV prevalence in specific groups [30]. The most recent weighted-average estimate of the number of HIV-infected persons in France using this “direct” method, from 1997, is 105,800 [30]. To estimate the number of patients living with HIV in 2005, we added 7,360 new HIV diagnoses per year [31] and subtracted 1,700 HIV-related deaths per year [32]. We determined that in 2005, 116,500–151,100 people in France were living with HIV, of whom only 77,400 were in care [33]. With the back-calculation approach, the 2005 prevalence of undiagnosed HIV infection was 0.10%, while with the direct method it was 0.18%. We used the more conservative estimate of 0.10% in our base case analysis (Table 1). When we considered the number of new HIV diagnoses in 2005, the incubation time from HIV infection to AIDS, and the mean delay from infection to initiation of care [20], [34], HIV incidence in the general population was estimated at 0.01/100 person-years (PY) (Table 1).

The prevalence of undiagnosed HIV in sub-populations and French Guyana ranged from 0.04% among heterosexuals to 6.17% among IDU. Incidence ranged from 0.01/100PY among heterosexuals to 0.99/100PY among MSM (see Appendix S1).

#### Delay from infection to HIV care

In 2005, mean CD4 count at initiation of HIV care under “current practice” conditions in French Hospitals, which included routine clinic visits and laboratory monitoring, was 372/µl (standard deviation, 257/µl), and 25% of patients initiated care at CD4 counts <200/µl (interquartile range, 200–500) (Table 1) [35]. We used the Screening and Disease Modules to estimate the mean time from HIV infection to initiation of HIV care in the “current practice” scenario, and found a mean delay from infection to linkage of 36 months, or a 2.8% monthly probability of non-routine testing, diagnosis and HIV care initiation (Table 1). This rate of non-routine HIV testing includes imperfect test results as well as imperfect linkage to care. The delay from HIV infection to diagnosis in the HIV screening scenarios was determined by the model and depended on screening frequency. In our analysis, when patients were offered routine HIV tests, an estimated 79% of patients accepted them and 75% of those who tested positive effectively linked to care [36], [37]. We assumed that patients who linked to care did so in the month after detection.

#### Test characteristics and frequencies

A routine HIV test, which included pre-test counseling, a blood draw, and a fourth-generation enzyme-linked immunosorbent assay (ELISA) test, cost €43 [19], [38]. Reactive tests were followed by a physician consult, a blood draw, and a confirmatory Western blot analysis (Table 1). The physician consult included post-test counseling and linkage to care (€22). Quality of life was reduced by 32% for seven days after a positive ELISA test, to account for the anxiety related to waiting for confirmation or refutation of a reactive test (Appendix S1, Table A1) [39]. We conducted extensive sensitivity analyses on these parameters.

### Secondary transmission

Recognizing that ART reduces infectivity by lowering HIV RNA levels [40], we determined the impact of earlier HIV diagnosis on secondary transmission by estimating the number of secondary cases per infected individual in each testing scenario (see Appendix S1). We used model output to obtain the number of life-months that treated and untreated patients spent in each of the model's HIV RNA strata. These values were multiplied by international data on transmission by HIV RNA level to obtain the total number of secondary transmissions [40]. Secondary transmission rates ranged from 0.2/100PY at HIV RNA levels <400 copies/ml to 9.0/100PY at HIV RNA levels >50,000 copies/ml (Table 1). We varied these input parameters in sensitivity analysis to account for the impact of HIV status knowledge on high-risk behavior.

Each secondary infection was assigned a survival loss and an economic cost. These were derived by comparing model-based estimates of quality-adjusted survival and lifetime medical costs among HIV-infected persons, assuming current standards of care and HIV-specific quality of life weights, to quality-adjusted survival and lifetime medical costs among HIV-uninfected persons [41]–[43]. In the “current practice” scenario, mean loss per secondary infection was 20.41 quality-adjusted life-months (QALM), discounted to the time of infection, and the mean additional cost of one secondary HIV infection was €100,150. We accounted for delays from primary to secondary HIV infection and from secondary HIV infection to diagnosis [7], [44].

### Sensitivity analyses

**Figure 1:**
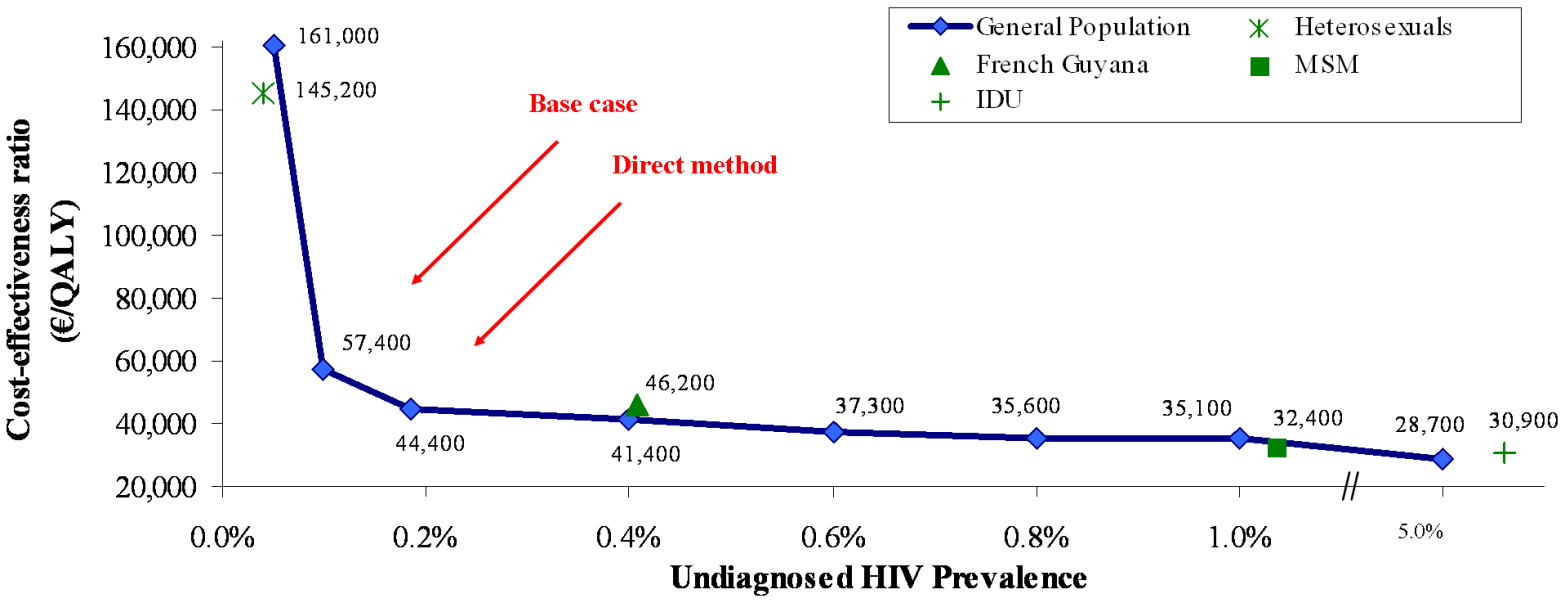
Effect of undiagnosed HIV prevalence on the cost effectiveness a one-time routine, voluntary HIV test vs. “current practice”, with base case incidence. Incidence rates are as follows: general population, 0.01/100PY; heterosexuals, 0.01/100PY; French Guyana, 0.35/100PY; MSM, 0.99/100PY; and IDU, 0.17/100PY. MSM: men who have sex with men; IDU: injection drug users; PY: person-year.

**Figure 2:**
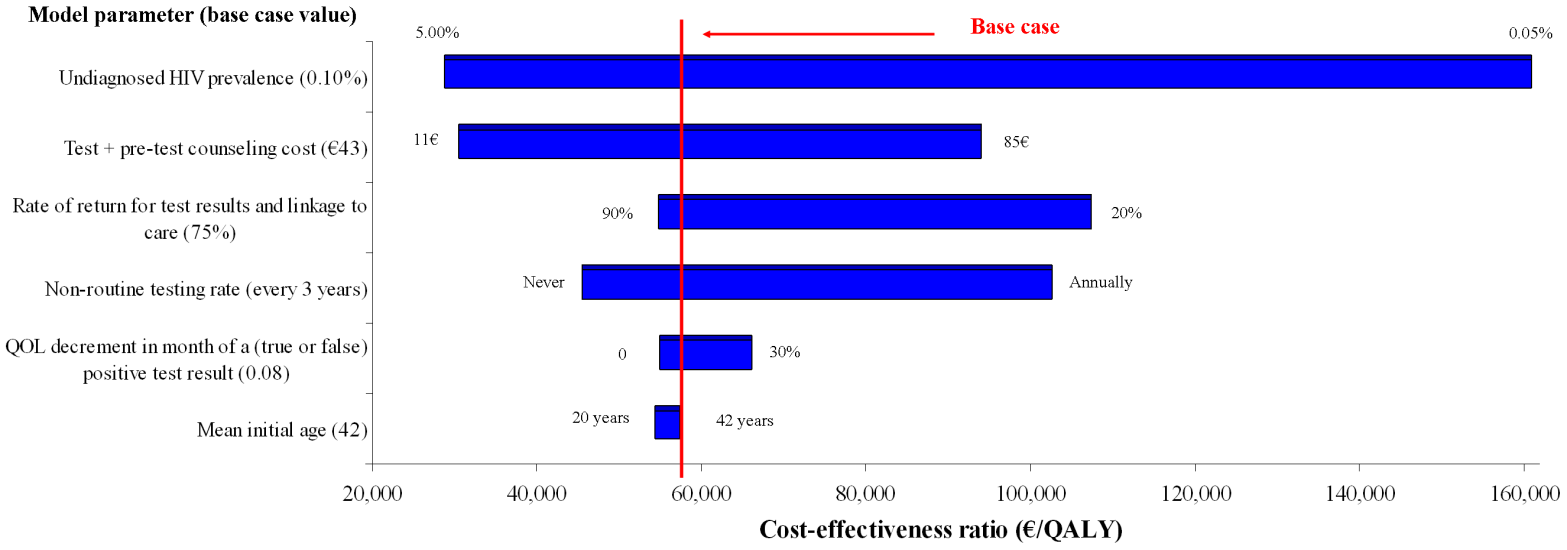
Sensitivity analyses: One-time routine, voluntary HIV test vs. “current practice” in the general population, with base case prevalence and incidence. The width of the bar indicates the variation in the incremental cost-effectiveness ratio associated with alternative parameter values for that input. The numbers to the right and left of the bars indicate the lower- and upper-bounds of the ranges used in sensitivity analyses.

We performed extensive sensitivity analyses on estimates of undiagnosed HIV prevalence, incidence, mean initial age, test acceptance, linkage to care, non-routine testing rates, HIV treatment costs, and the quality of life decrement associated with a reactive test (Figures 1 and 2). We also varied the cost of the HIV screening program. In addition to varying the cost of the HIV test, we added the fixed cost of putting in place new routine screening programs throughout France. These start-up costs would mainly consist of training general practitioners. We assumed that 80% of the 103,916 general practitioners in France would participate in a two-day training course, each comprising 20 physicians [45]. As part of the training, physicians would receive €330 per day and instructors would receive €1,500 per course; overhead costs would be €2,000 per course [46]. Overall start-up costs were thus estimated at €69,415,900. When we considered that 10 to 20 million people – 25–50% of the targeted population – would receive HIV tests, program start-up costs added €3.47–6.94 to the per person cost of routine HIV screening. Finally, we varied ART initiation criteria and ART regimen efficacies, and considered a zero probability of transmission in patients with HIV RNA <500 copies/ml and increases in high-risk behavior upon HIV diagnosis and ART initiation (Appendix S1, Table A3).

### Ethics Statement

The medical ethics review committees of the contributing hospitals exempted this research from institutional review board approval, because all input data were obtained from secondary sources, we did not have access to any patient identifiers, and there was no direct contact with any human subjects.

## Results

### Base case analysis

In the “current practice” scenario, mean CD4 count at diagnosis was 308/µl among prevalent cases and 370/µl overall; 25% of patients were diagnosed after presenting to care with an AIDS-defining opportunistic disease (Appendix S1, Table A2). Mean discounted life expectancy for HIV-infected patients was 242.82 QALM (419.00 undiscounted QALM) (Table 2). When we took secondary HIV cases into account, mean discounted life expectancy in the general population was 268.77 QALM (479.12 undiscounted QALM) and per person discounted costs were €1,290.

**Table 2:** Routine, voluntary HIV screening in the French general population.

Variable		“Current practice”	“Current practice” and screen once	“Current practice” and screen every 5 years	“Current practice” and screen annually
**Reduction in secondary HIV cases, %** a		--	7.5	10.3	17.8
**HIV-infected persons**					
	Mean undiscounted life expectancy, months	451.46	453.84	456.82	459.87
	Mean undiscounted quality-adjusted life expectancy, QALM	419.00	421.18	423.97	426.83
	Mean discounted life expectancy, months	258.34	259.73	260.85	262.14
	Mean discounted quality-adjusted life expectancy, QALM	242.82	244.09	245.14	246.36
	Mean discounted lifetime costs per person, 2007 €	134,880	138,320	142,080	148,190
**Population**					
	Mean undiscounted life expectancy, months	479.13	479.15	479.17	479.20
	Mean undiscounted quality-adjusted life expectancy, QALM	479.12	479.14	479.16	479.17
	Mean discounted life expectancy, months	268.83	268.84	268.85	268.86
	Mean discounted quality-adjusted life expectancy, QALM	268.77	268.78	268.78	268.78
	Mean discounted lifetime costs per person, 2007 €	1,290	1,340	1,500	2,130
**Incremental cost-effectiveness** b					
	Only 1° HIV cases, €/QALY	--	61,100	443,700	Dominated c
	1° and 2° HIV cases, €/YLS	--	51,500	215,500	737,000
	1° and 2° HIV cases, €/QALY	--	57,400	332,200	Dominated c

QALM: quality-adjusted life-month; QALY: quality-adjusted life-year; YLS: year of life saved.

a Reduction in secondary cases is compared to “current practice” at 10 years.

b Incremental cost-effectiveness  =  (difference in cost) / (difference in quality-adjusted life expectancy), where the comparator is always the next smallest, not dominated, alternative.

c A dominated strategy has a higher cost and an equal or lower quality-adjusted life expectancy than some combination of other strategies.

When we added a one-time routine HIV test to “current practice,” mean CD4 count at diagnosis increased to 362/µl among prevalent cases and 379/µl overall. The proportion of patients diagnosed with HIV after presenting to care with an AIDS-defining opportunistic disease decreased to 23% (Appendix S1, Table A2). Secondary cases at ten years decreased by 7.5% (Table 2). One-time routine HIV screening conferred an additional 1.27 discounted QALM per HIV-infected person (2.18 undiscounted QALM) and an additional 0.01 QALM/person in the general population (0.02 undiscounted QALM), at an additional cost of €50/person. When we did not account for the impact of HIV screening on secondary transmission, the cost-effectiveness of adding one routine HIV test to “current practice” was €61,100 per quality-adjusted life-year (QALY). When we combined the favorable effects of screening on HIV transmission with individual-level life expectancies and costs, the cost-effectiveness ratio improved to €57,400/QALY. Increasing the frequency of screening to once every five years in the general population cost €332,200/QALY compared to one-time routine HIV screening. Screening annually further increased costs but did not produce any health benefits, because the quality of life losses associated with false-positive tests offset any survival gains.

### Sensitivity analyses

When we kept incidence constant, variations in the prevalence of undiagnosed HIV infection had an impact on results (Figure 1). When the prevalence of undiagnosed HIV decreased from 0.10% to 0.05%, the cost-effectiveness ratio of one-time routine HIV screening compared to “current practice” increased to €161,000/QALY. When we increased the prevalence of undiagnosed HIV in France to 0.18% (from the “direct method”), the cost-effectiveness of a one-time routine HIV test compared to “current practice” was €44,400/QALY.

We performed a similar analysis to determine the cost-effectiveness of routine HIV screening among IDU, MSM and heterosexuals, as well as in French Guyana (Figure 1, Table 3). Among IDU and in French Guyana, annual screening led to cost-effectiveness ratios of €51,200 and €46,500/QALY, respectively, compared to screening every 5 years. Adding a one-time routine HIV test to current practice among MSM increased life expectancy by 0.08 QALM and increased mean costs by €210, leading to a cost-effectiveness ratio of €32,400/QALY compared to current practice. Screening annually led to higher survival and costs, but a less favorable incremental cost-effectiveness ratio (€97,200/QALY) compared to a one-time HIV test. Among heterosexuals, adding a one-time routine HIV test to current practice led to a cost-effectiveness ratio of €145,200/QALY. Screening annually was more expensive and produced no health benefit.

**Table 3:** Routine, voluntary HIV screening among French sub-populations ^a^.

Variable		“Current practice”	“Current practice” and screen once	“Current practice” and screen every 5 years	“Current practice” and screen annually
**Injection drug users (undiagnosed prevalence, 6.17%; incidence, 0.17/100 PY)**					
	Mean undiscounted life expectancy, months	452.71	453.79	454.37	455.21
	Mean discounted quality-adjusted life expectancy, QALM	258.62	259.30	259.51	259.88
	Mean discounted lifetime costs per person, 2007 €	27,480	29,240	29,960	31,540
	Incremental cost-effectiveness, €/QALY	--	30,900	41,200	51,200
**French Guyana (undiagnosed prevalence, 0.41%; incidence, 0.35/100 PY)**					
	Mean undiscounted life expectancy, months	455.71	455.77	457.11	458.41
	Mean discounted quality-adjusted life expectancy, QALM	262.45	262.50	262.91	263.28
	Mean discounted lifetime costs per person, 2007 €	21,980	22,170	23,100	24,510
	Incremental cost-effectiveness, €/QALY	--	Dominated b	28,800	46,500
**Men who have sex with men (undiagnosed prevalence, 1.70%; incidence, 0.99/100 PY)**					
	Mean undiscounted life expectancy, months	391.68	391.80	391.94	392.44
	Mean discounted quality-adjusted life expectancy, QALM	241.48	241.56	241.58	241.69
	Mean discounted lifetime costs per person, 2007 €	57,530	57,750	58,000	58,840
	Incremental cost-effectiveness, €/QALY	--	32,400	Dominated b	97,200
**Heterosexual population (undiagnosed prevalence, 0.04%; incidence, 0.01/100 PY)**					
	Mean undiscounted life expectancy, months	479.82	479.83	480.06	479.86
	Mean discounted quality-adjusted life expectancy, QALM	268.98	268.98	268.98	268.98
	Mean discounted lifetime costs per person, 2007 €	580	630	770	1,400
	Incremental cost-effectiveness, €/QALY	--	145,200	963,000	Dominated b

PY: person-year; QALM: quality-adjusted life-month; QALY: quality-adjusted life-year

a All results incorporate the favorable effects of routine HIV screening on secondary HIV transmission. The cost-effectiveness results shown are not calculable, due to rounding.

b A dominated strategy has a higher cost and an equal or lower quality-adjusted life expectancy than some combination of other strategies.

We evaluated changes in the cost-effectiveness of one-time, population-wide, routine HIV screening when we varied major model parameters, one at a time, over a range of plausible values (Figure 2). Variations in HIV test cost and rates of undiagnosed HIV prevalence, linkage to care and non-routine testing had the largest impact on results. If the quality of life decrement associated with waiting for confirmation or refutation of a reactive test decreased, a one-time HIV test in the general population became more attractive compared to “current practice.” Finally, when we reduced the mean age of the cohort but maintained the base case prevalence, one-time routine HIV screening became more cost-effective.

New HIV incidence rates, which account for very recent HIV infections, were recently derived for the French general population and sub-populations [47]. These estimates were similar to ours and did not have an impact on our main results. Results also remained robust to variations in HIV test acceptance rate, HIV test sensitivity and specificity, ART efficacy and initiation criteria, HIV screening start-up costs, and secondary transmission rates (Appendix S1, Table A3).

## Discussion

We found that a one-time routine, voluntary HIV test in the French general population decreases the delay from HIV infection to diagnosis, increases mean CD4 count at diagnosis, improves survival among HIV-infected patients, reduces secondary infections at ten years, and achieves cost-effectiveness ratios that are viewed as acceptable by French standards [48]. More frequent screening is economically justifiable in specific sub-populations that are at higher risk for HIV, such as MSM, IDU, and the population of French Guyana.

This study is the first to evaluate the cost-effectiveness of routine HIV screening in Europe. Previous studies have found the cost-effectiveness of one-time routine HIV screening in the United States to range from less than $50,000/QALY (€41,400/QALY in 2007 €) in health care settings with undiagnosed HIV prevalence rates >0.05% [49], to $60,700/QALY (€50,200/QALY, in 2007 €) in populations with an undiagnosed HIV prevalence of 0.10% [7], [8]. Several factors may lead to higher cost-effectiveness ratios in the European setting, including higher rates of risk-factor-based, non-routine HIV testing, higher CD4 counts at diagnosis, and fewer patients presenting to care with AIDS-defining opportunistic diseases [42], [50]. Still, roughly one-third of HIV-infected individuals in France are unaware of their HIV status [4] and the prevalence of undiagnosed HIV is estimated at 0.10%. Recent evidence shows that delayed diagnosis in France is more common among patients at a perceived low risk of infection, such as heterosexuals and older individuals [5], because current HIV testing strategies specifically target high-risk groups, namely MSM and IDU. Although HIV screening in France is slightly less cost-effective than in the US, primarily due to the lower prevalence of undiagnosed HIV in France, one-time HIV screening compares favorably to other screening interventions recommended in Western Europe (Table 4) [51]–[55].

**Table 4:** Cost-effectiveness of common and accepted screening interventions recommended in Europe.

Screening programs	Cost-effectiveness a	Country of analysis	Source
Cervical cancer screening every 5 years, women aged 25–65 years	€2,200/YLS	France	[51]
Rectal cancer screening by fecal occult blood test every 2 years, men and women aged 50–74 years	€3,700/YLS	France	[52]
Breast cancer screening every 2 years by mammogram, women aged 50–65 years	€23,300/YLS	France	[53]
Annual Chlamydia screening, men and women aged <25 years b	€43,100 – 318,500/QALY^a^	England	[54]
One-time hepatitis C screening and treatment, prisoners	€86,800/QALY	England/Wales	[55]
One-time hepatitis C screening and treatment, prisoners aged >35 years	€203,100/QALY	England/Wales	[55]

YLS: years of life saved; QALY: quality-adjusted life-year.

a All costs updated to 2007 €.

b Cost-effectiveness varies depending on the probability of pelvic inflammatory disease after Chlamydia infection.

The cost-effectiveness of routine HIV screening estimated in this study may apply to other European countries. One-time HIV screening was associated with favorable cost-effectiveness ratios when undiagnosed HIV prevalence rates were ≥0.10%. Although undiagnosed HIV prevalence varies by region, several European countries have reported that HIV-infected individuals frequently present to care with AIDS-defining symptoms [5], [56]–[59]. Recent studies have estimated that 30% of HIV-infected individuals in the European Union remain undiagnosed, with proportions ranging from 12–20% in Denmark, Norway and Sweden, to over 50% in Poland [60]. In Western European countries such as Italy, Spain or Switzerland where the prevalence of diagnosed HIV is equal to or higher than in France [61], it is likely that the prevalence of undiagnosed HIV is above 0.10% and that rates of non-routine HIV testing and CD4 counts at diagnosis are no higher than in France.

Variations in several parameters led to important results. First, the frequency of routine HIV screening was strongly dependent on the targeted sub-population. More frequent screening was associated with favorable cost-effectiveness ratios among sub-populations with higher HIV incidence rates, such as IDU and French Guyana. The cost-effectiveness of annual screening in MSM was higher than in IDU and French Guyana, because non-routine testing rates and CD4 counts at HIV diagnosis in this group are already high. Second, the attractiveness of routine screening hinges on earlier and more frequent presentation to care among persons who test positive for HIV. Interventions to improve linkage to care should be implemented alongside routine, voluntary HIV screening, particularly in marginalized groups known to be at risk for delayed presentation to care and loss to follow-up, such as immigrants [58]. Third, we recognize that the population-level data we employed to model secondary HIV transmission may under-represent rates of infection in high-risk sub-populations. While we deliberately chose conservative input values to understate our cost-effectiveness findings, sensitivity analysis revealed that secondary transmission rates had a smaller impact on results than anticipated. This is because test acceptance and linkage to care rates were imperfect, ART initiation was not immediate, ART failure could occur as a result of non-adherence and/or toxicity, and results were discounted for the passage of time to secondary infection.

This analysis has several limitations. The CEPAC model combines data from multiple sources and relies on various assumptions to estimate the long-term benefits of alternative routine HIV screening strategies. First, undiagnosed HIV prevalence and incidence rates were estimated from back-calculations [30], [34]. Second, in the absence of other data, the 2.8% monthly probability of diagnosis and linkage via non-routine testing was estimated from reported CD4 counts at initiation of care in France [35]. Third, we assumed that the anxiety associated with waiting for confirmation or refutation of a reactive HIV test in France was similar to the US [39]. Fourth, we were not able to evaluate the cost-effectiveness of routine HIV screening in immigrant populations, for whom HIV prevalence and incidence rates are high, because data on this sub-population are scarce. Fifth, although we did account for the effect of HIV RNA levels on transmission, we did not incorporate the behavioral effects of counseling or the role of ART in prolonging infectious survival and possibly increasing sexual risk-taking. However, most of these assumptions were largely conservative with respect to the benefits of routine HIV screening.

New strategies that encourage earlier HIV testing in France are needed. This study suggests that one-time routine, voluntary HIV screening should be implemented on a population-wide basis in France. More frequent screening is warranted in sub-populations with high HIV prevalence and incidence rates. These screening strategies will only be successful if efforts to increase both the acceptability of HIV screening and linkage to care are implemented.

## Supporting Information

Appendix S1 Technical Appendix.
(0.52 MB DOC)
