# Supplementary material for: Routine HIV Screening in France: Clinical Impact and Cost-Effectiveness
Source: PLoS One. 2010 Oct 1;5(10):e13132. doi: 10.1371/journal.pone.0013132 (PMC2956760; doi:10.1371/journal.pone.0013132)
Supplement: Appendix S1 — Technical Appendix. (0.52 MB DOC) [file pone.0013132.s001.doc]

**Routine HIV screening in France: Clinical impact and cost-effectiveness**

Appendix S1

**Introduction**

The analysis described in the main manuscript is built upon the foundation of the Cost-Effectiveness of Preventing AIDS Complications (CEPAC) Model, a computer simulation of the clinical management and economics of HIV disease. The CEPAC Model comprises two distinct pieces: a Disease Module, which simulates the natural history of HIV illness and the impact of alternative therapies in an infected individual; and a Screening Module, which simulates the prevalence, incidence, detection, and referral to care of infected persons in an at-risk population.

Ideally, the questions posed in this paper would be addressed by conducting large-scale, randomized clinical trials that could produce excellent estimates of infection times, non-AIDS death times, and CD4 counts at diagnosis for a range of alternative target populations, geographic settings, testing frequencies, and HIV screening policies. However, clinical trials and observational studies in HIV disease can only be conducted over limited time horizons, using a small number of alternative interventions, and at substantial cost. We therefore turn to a simulation model and “what-if” analysis to perform formal comparisons using secondary data analysis, extrapolate to longer time horizons, and consider the full range of geographic settings and target populations of interest to policy makers. Our Monte Carlo simulation model characterizes the course of disease in a given patient as a sequence of probabilistic transitions from one health state to another. While any given patient’s experience may be seen as arbitrary*,* large numbers of these individual simulations are aggregated to develop stable estimates of outcomes.

In this Technical Appendix, we provide greater detail on the mechanics of both the Disease Module and the Screening Module. We also provide details on our estimates of undiagnosed HIV prevalence and incidence in French sub-populations, and our efforts to estimate the impact of expanded routine, voluntary HIV screening on secondary HIV transmission.

**Disease Module**

We used a widely cited, computer-based, state-transition, Monte Carlo simulation model of the progression and outcomes of HIV disease in a hypothetical cohort of patients [1-5]. The Disease Module characterizes the natural history of illness in each individual patient as a sequence of monthly transitions from one “health state” to another and a random number generator and set of estimated probabilities determine the sequence of movements between health states. Each patient’s clinical course is followed from the time of entry into the module until death. A running tally is maintained of all clinical events, the length of time spent in each health state, and the costs and quality-of-life associated with each health state. Upon the patient’s death, summary statistics are recorded and a new patient enters the model. The model typically achieves statistical convergence for overall performance measures such as average life expectancy, quality-adjusted life expectancy, and medical costs with sample sizes of 5 million.

Health states describe the patient’s current health, relevant history, quality-of-life, and resource utilization patterns. They predict clinical prognosis, including disease progression, immune system deterioration, development and relapse of different opportunistic diseases, toxic reactions to medications, resistance to therapy, and mortality. The Disease Module defines three general categories of health states: chronic states, opportunistic diseases, and death. Most of the time, patients reside in one of the chronic states, where progression of disease and immune system deterioration (CD4 decline) take place. Patients who develop complications (e.g., opportunistic diseases or drug-related toxicities) temporarily move to the opportunistic disease health state, where quality-of-life is lower and resource consumption levels and mortality rates are higher. Death can occur from either a particular opportunistic disease, chronic AIDS (e.g., wasting), or non-AIDS-related causes in either a chronic state or during an opportunistic disease.

Chronic health states and opportunistic diseases are stratified by: current and nadir CD4 cell count (>500 cells/mm^3^; 301–500 cells/mm^3^; 201–300 cells/mm^3^; 101–200 cells/mm^3^; 51–100 cells/mm^3^; <50 cells/mm^3^) and current and set-point HIV RNA level (>100,000 copies/ml; 30,001–100,000 copies/ml; 10,001–30,000 copies/ml; 3,001–10,000 copies/ml; 501–3,000 copies/ml; <500 copies/ml). Upon entry into the Disease Module, a patient is randomly assigned to a health state based upon a set of user-specified probability distributions. By permitting the user to define initial population distributions for patient age, sex, CD4 cell count, HIV RNA, and other demographic and clinical attributes, the module has the flexibility to evaluate a broad range of different patient cohorts. We used a normal distribution for continuous variables such as age and CD4 cell count, and a binomial distribution for categorical variables such as sex.

At the start of each one-month cycle, the Disease Module records the patient’s CD4 cell count, HIV RNA level, history of opportunistic diseases, and current therapies to determine the probabilities that indicate movement to a new state in the subsequent month. Key parameters of the natural history of HIV disease, including rates of disease progression, opportunistic disease and mortality, as well as the effects of therapy, are derived from clinical cohorts and public use datasets [6-17]. HIV RNA levels determine the rate at which the CD4 counts decline [7]. Patients with a history of opportunistic disease have a higher risk of recurrence, depending on CD4 count and current antiretroviral therapy (ART) use.

Patients can receive up to six sequential ART regimens. First-line ART consists of tenofovir and emtricitabine plus efavirenz and second-line ART consists of boosted atazanavir plus two nucleoside reverse transcriptase inhibitor (NRTI) [8,9]. Once patients start third-line therapy, genotype tests generally determine individualized regimens. Subsequent lines of ART are therefore modeled as generic regimens with wide ranges of efficacy, represented by various recent studies [9-12,18-24]. Patients who initially succeed on ART have a monthly probability of virologic failure. Patients are considered to have failed a regimen after a repeated observed increase in HIV RNA level [25]. The Disease Module accounts for a 12-month lag between virologic failure and CD4 decline.

In addition to life expectancy, quality-adjusted life expectancy, and total lifetime costs, model output may include disaggregated estimates of average cost per patient (e.g., drugs, laboratory tests, hospitalizations), performance of therapy (e.g., time on therapy, time to viral load rebound, rates of toxicity), and morbidity (specific opportunistic disease incidence rates, and causes of death).

Input data for the Disease Module have been described and published previously [3,6,26]. For the reader’s convenience, selected data are reproduced in Table A1.

**Screening Module**

The Screening Module simulates the epidemiology and detection of HIV infection in an at-risk population (Figure A1). It accounts for such parameters as HIV prevalence and incidence; HIV test sensitivity, specificity, and cost; screening frequency; and rates of test acceptance and linkage to care. This information is conveyed to the Disease Model, where it is linked to output on the timing of untreated AIDS-defining complications to establish whether, when, and how an individual case of HIV infection is first detected.

Figure A1 provides a conceptual overview of the Screening Module. Individual members of the population enter the simulation, one at a time. A random-number generator makes use of “time-to-event” probability distributions to assign each incoming person a unique set of four HIV-related event times: (1) time of HIV infection, t_i_; (2) time of next HIV test performed within the context of a specific routine HIV screening program, t_p_; (3) time of next non-routine HIV test (such as an HIV test performed in an office or clinic setting), t_b_; and (4) time of non-AIDS-related death, t_d_.

User-specified HIV incidence is a hazard rate which, in the absence of reliable data, is assumed to be constant over time. Based on the monthly probability of infection, a random number generator determines the time to infection for each patient. Similarly, non-HIV-related mortality is based on standard life tables stratified by gender and age [27]. When a patient’s time to non-HIV-related death exceeds the time to infection, infection occurs during the patient’s lifetime.

In the large majority of instances, t_i_ far exceeds t_d_, reflecting the reality that most people die uninfected. A simple “IF/THEN” statement (represented by the upper diamond in Figure A-1) makes this determination; such cases will never enter the Disease Module. For some individuals, however, t_i_<t_d_ and HIV infection occurs during their lifetimes. At the given time t_i_, these patients proceed to the Disease Module, which transcribes their age at the moment of infection and begins simulating the progress of their illness and associated clinical and economic outcomes. These individuals are not eligible to receive any kind of HIV therapy within the Disease Module until and unless their HIV infection is identified. Thus, the Disease Module simulates disease progression for all infected individuals, but only detected cases are eligible for ART and opportunistic disease prophylaxis. Patients who are successfully detected and linked to care are assumed to receive services (and incur associated costs) that conform to national guidelines. These include CD4 count and HIV RNA laboratory monitoring every three months, as well as ART and opportunistic disease prophylaxis at the appropriate CD4 cell count thresholds [25].

Detection of HIV infection can occur via one of three discrete mechanisms. First, an infected individual can receive an HIV-positive test result within the context of a specific routine screening program (t_b_>t_p_>t_i_). Second, an infected individual can receive an HIV-positive test result within the context of a non-routine testing mechanism (t_p_>t_b_>t_i_). Third, an infected individual can be detected after seeking medical care for an AIDS-defining illness. The Screening Module determines the time of detection via the first two mechanisms, using a random number generator to combine user-specified assumptions regarding HIV test accuracy, program cost, and rates of test acceptance, return for results, linkage-to-care, and non-routine testing, as obtained from national French data (Table A1). This information is conveyed to the Disease Module which, in turn, determines the actual time of detection by comparing the time of screen-detection to the time of the first OI. In our base case analysis, mean CD4 count among patients with undiagnosed asymptomatic HIV infection was 439/µl (standard deviation, 257/µl), which is equivalent to the mean CD4 count among non-AIDS HIV-infected patients who initiated care in 2005 [28]. We varied this input in sensitivity analysis (Table A3).

The Screening Module defines four distinct HIV states: HIV-negative, HIV-acute, HIV-asymptomatic, and HIV-symptomatic. The duration of the acute HIV phase in the CEPAC model determines the window during which the ELISA test is less sensitive and thus less likely to detect HIV. Because the new combined HIV-1/HIV-2 antigen-antibody tests that are currently used in France are highly sensitive beginning one month after infection [29], we considered that newly infected patients remained in the HIV-acute state for one month before entering the chronic, HIV-asymptomatic state [30]. Users can specify both pre- and post-seroconversion test sensitivities and specificities to capture the “window” properties of alternative test protocols.

Each individual is tracked from the time of entry into the Screening Module until death or transition to the Disease Module. The Screening Module tracks test offers and acceptances, rates of return for test results and linkage to care, and all costs and quality-of-life effects. Upon each patient’s departure from the Screening Module, summary statistics are recorded and a new patient simulation begins. Sample size requirements depend upon user-specified prevalence, incidence, and testing program performance. Outputs of the Screening Module include: total program enrollment by HIV status; test acceptance and return rates by HIV status; frequency of testing and total number and type of tests performed; total true- and false-positive tests and true- and false-negative tests; CD4 count and HIV RNA at detection; time from infection to diagnosis; mechanism of detection; and total testing-related costs.

**Prevalence and Incidence**

Input data for the Screening Module including the methods used for estimating the undiagnosed HIV prevalence in the general population are described in the main manuscript. We performed similar analyses to determine 2005 prevalence and incidence rates among men who have sex with men (MSM), injection drug users (IDU) and heterosexuals in France.

Using a “back-calculation” method, which incorporates data on the reported number of AIDS cases, incubation time from HIV infection to AIDS [31], and efficacy of ART, we estimated that the number of HIV-infected MSM in 2000 was 31,100 [32], or 35% of the overall HIV-infected population in France. We assumed that the proportion of MSM among undiagnosed HIV-infected persons was also 35%. The number of undiagnosed HIV-infected MSM in 2005 was thus 13,770. The MSM population was estimated to be 808,610, or 4.1% of the French male population aged 18-69 in France [33,34], leading to a prevalence of undiagnosed HIV among MSM of 1.70% in 2005. To estimate the HIV incidence rate among MSM, we used a similar extended back-calculation that accounted for the number of new HIV diagnoses in 2005, the incubation time from HIV infection to AIDS and the delay from infection to linkage to care. In a population of 315,560 active MSM and with an estimated 3,115 new infections in 2005, HIV incidence was estimated to be 0.99/100 person-years (PY) [33,35]. Because MSM generally present to care earlier than the general population [36], we may have overestimated prevalence, but this is not likely to have affected our results substantially. This input was varied in sensitivity analysis (Table A3).

We used a similar method to estimate the undiagnosed HIV prevalence among IDU. With the “back-calculation” method, the number of HIV-infected IDU was estimated to be 20,200 in the year 2000, or 23% of the overall HIV-infected population in France [32]. Assuming that the proportion of IDU among undiagnosed HIV patients was also 23%, 8,940 IDU had undiagnosed HIV infection in 2005. In a population of 145,000 persons in France aged 15-64 who report having used injection drugs at least once [37], the prevalence of undiagnosed HIV was 6.17% in 2005. The number of new infections among active IDU in 2005 was 134 in a population of 81,000, leading to an incidence of 0.17/100PY [35,37]. Because IDU generally present to care late [36], we may have underestimated prevalence, but this is not likely to have affected our results substantially. This input was varied in sensitivity analysis (Table A3).

We assumed the number of HIV-infected heterosexuals and the size of the heterosexual population was equivalent to the general population minus the MSM and IDU sub-populations, and found that the undiagnosed prevalence of HIV among heterosexuals was 0.04% in 2005. Similarly, the number of new infections among heterosexuals in 2005 was equivalent to the new infections in the general population minus those in the MSM and IDU subpopulations, leading to an incidence of 0.01/100PY.

Because similar data were not available for French Guyana, we used data on pregnant women to derive the prevalence of undiagnosed HIV. We extrapolated the 1.3% prevalence of diagnosed HIV among pregnant women to the entire population of French Guyana, where only 1,000 patients were in care in 2005 [38]. The prevalence of undiagnosed HIV in a population of 112,030 was 0.41%. The number of new infections in French Guyana was 392, according to an unpublished InVS analysis, leading to an incidence of 0.35/100PY.

**Estimating Secondary Infections**

The success of ART and behavioral counseling in reducing transmission of HIV infection can be gauged by the reductions in secondary infections and increases in life expectancy they produce. In order to estimate the number of secondary cases in the “current practice” scenario and each HIV screening scenario, we first determined the total number of person-months spent in each HIV RNA stratum by month, starting from each patient’s entry into the model. These values were multiplied by monthly probabilities of transmission by HIV RNA level, derived from published international transmission rates [39], to find the number of secondary infections per month. To determine the total number of secondary infections in the cohort, we added the monthly secondary infections.

We then determined increased costs and decreased life expectancy associated with secondary infections, discounted to the time of detection. We used the Disease Module to estimate the average cost [26], life expectancy and quality-adjusted life expectancy for HIV-infected individuals with the same characteristics as those detected with the Screening Module. We compared the medical costs, life expectancy and quality-adjusted life expectancy of HIV-infected patients to those of HIV-uninfected individuals, using standard French life tables and United States quality of life data. We discounted these values first to the time of infection, using the derived monthly secondary infections described above, and then to the time of detection. We estimated that mean time from infection to detection was three years, as described in the main text.

Given the uncertainty surrounding the effect of ART on secondary transmissions, we consider a range of plausible scenarios. We varied the effect of ART on transmission from half to double base case values, and we performed an analysis in which we assumed no transmissions occurred at HIV RNA levels <500 copies/ml.

**Results**

Table A2 shows additional results from the base case analysis. Table A3 shows the results of several sensitivity analyses that are not included in the main text.

**References**

| **Table A1. Selected Disease and Screening Module input data** | | | | | | | | | | | | | |
| --- | --- | --- | --- | --- | --- | --- | --- | --- | --- | --- | --- | --- | --- |
| **Variable** | | | | | | | | | | **Value** | | | **Reference** |
| **ELISA test characteristics (sensitivity analysis range)** | | | | | | | | | | | | | |
|  | | Sensitivity pre-infection and <1 month post-infection, % | | | | | | | | 1.0 (0 – 5.0) | | | [40] |
|  | | Sensitivity >1 month post-infection, % | | | | | | | | 100 (97 – 100) | | | [40] |
|  | | Specificity, % | | | | | | | | 99 (95 – 100) | | | [40] |
| **QOL decrement in month of true or false positive test (sensitivity analysis range)** | | | | | | | | | | 0.08 (0 – 0.30) | | | [41] |
| **Viral load distribution at initiation of HIV care (copies/ml), %** | | | | | | | | | | | | | |
|  | | | | | Patients without AIDS | | | | Patients with AIDS | | | |  |
|  | >100,000 | | | | 18.8 | | | | 43.9 | | | | [28] |
|  | 30,001 – 100,000 | | | | 21.1 | | | | 20.3 | | | | [28] |
|  | 10,001 – 30,000 | | | | 15.2 | | | | 5.4 | | | | [28] |
|  | 3,001 – 10,000 | | | | 13.9 | | | | 6.1 | | | | [28] |
|  | 501 – 3,000 | | | | 11.2 | | | | 6.1 | | | | [28] |
|  | <500 | | | | 19.8 | | | | 18.2 | | | | [28] |
| **Mean monthly decline in CD4 count by HIV RNA level (copies/ml), cells/µl (SD)** | | | | | | | | | | | | | |
|  | | >30,000 | | | | | | | | 6.4 (0.3) | | | [7] |
|  | | 10,001 – 30,000 | | | | | | | | 5.4 (0.2) | | | [7] |
|  | | 3,001 – 10,000 | | | | | | | | 4.6 (0.2) | | | [7] |
|  | | 501 – 3,000 | | | | | | | | 3.7 (0.2) | | | [7] |
|  | | <500 | | | | | | | | 3.0 (0.3) | | | [7] |
| **Table A1. (continued)** | | | | | | | | | | | | | |
| **Variable** | | | | | | | | | | **Value** | | | **Reference** |
| **Monthly probability of opportunistic diseases by CD4 count (cells/µl), %** | | | | | | | | | | | | | |
|  | |  | | <50 | | 51-100 | 101-200 | 201-300 | | 301-500 | | >500 |  |
|  | | PCP | | 1.17 | | 0.35 | 0.35 | 0.17 | | 0.06 | | 0.06 | [6] |
|  | | MAC | | 0.52 | | 0.30 | 0.16 | 0.02 | | 0.02 | | 0.00 | [6] |
|  | | Toxoplasmosis | | 0.66 | | 0.14 | 0.25 | 0.13 | | 0.11 | | 0.03 | [6] |
|  | | CMV | | 0.87 | | 0.36 | 0.16 | 0.02 | | 0.09 | | 0.02 | [6] |
|  | | Fungal infections | | 0.94 | | 0.22 | 0.22 | 0.07 | | 0.09 | | 0.04 | [6] |
|  | | Other infections | | 2.37 | | 1.53 | 0.91 | 0.69 | | 0.34 | | 0.20 | [6] |
| **Mortality attributable to opportunistic diseases, %** | | | | | | | | | | | |  |  |
|  | | PCP | | | | | | | | 7.0 | | | [6] |
|  | | MAC | | | | | | | | 16.9 | | | [6] |
|  | | Toxoplasmosis | | | | | | | | 9.6 | | | [6] |
|  | | CMV | | | | | | | | 9.0 | | | [6] |
|  | | Fungal infections | | | | | | | | 0.0 | | | [6] |
|  | | Other infections | | | | | | | | 4.8 | | | [6] |
| **Costs, 2007 €** | | | | | | | | | |  | | |  |
|  | | Genotypic resistance test, /test | | | | | | | | 297 | | | [42] |
|  | | CD4 count test, /test | | | | | | | | 22 | | | [42] |
|  | | HIV RNA test, /test | | | | | | | | 59 | | | [42] |
|  | | | | | | | | | | | | | |
|  | | | | | | | | | | | | | |
| **Table A1. (continued)** | | | | | | | | | | | | | |
| **Variable** | | | | | | | | | | | **Value** | | **Reference** |
|  | | Antiretroviral regimens, /year | | | | | | | |  | | |  |
|  | |  | TDF/FTC + EFV | | | | | | | 8,950 | | | [43] |
|  | |  | ATV/r + 2 NRTIs | | | | | | | 11,140 | | | [43] |
|  | |  | 3^rd^-line | | | | | | | 13,430 | | | [43] |
|  | |  | 4^th^-line | | | | | | | 24,000 | | | [43] |
|  | |  | 5^th^-line | | | | | | | 29,280 | | | [43] |
|  | |  | 6^th^-line | | | | | | | 14,300 | | | [43] |
| **Health-related quality of life by CD4 count (cells/µl), % ^a^** | | | | | | | | | | |  | |  |
|  | | <50 | | | | | | | | | 83 | | [44] |
|  | | 51-200 | | | | | | | | | 85 | | [44] |
|  | | 201-500 | | | | | | | | | 86 | | [44] |
|  | | >500 | | | | | | | | | 87 | | [44] |
| **Health-related quality of life by opportunistic disease, % ^a^** | | | | | | | | | | |  | |  |
|  | | PCP | | | | | | | | | 74 | | [45] |
|  | | MAC | | | | | | | | | 69 | | [45] |
|  | | Toxoplasmosis | | | | | | | | | 69 | | [45] |
|  | | CMV | | | | | | | | | 78 | | [45] |
|  | | Fungal infections | | | | | | | | | 78 | | [45] |
|  | | Other infections | | | | | | | | | 69 | | [45] |
| ELISA: enzyme-linked immunosorbent assay; QOL: quality of life; PCP: *Pneumocystis* *jiroveci* pneumonia; MAC: *Mycobacterium avium* complex; CMV: cytomegalovirus; TDF: tenofovir; FTC: emtricitabine; EFV: efavirenz; ATV/r: ritonavir-boosted atazanavir; NRTI: nucleoside reverse transcriptase inhibitor.  ^a^ Health-related quality of life inputs are monthly quality of life adjustment factors that modify the current quality of life of the simulated patient. | | | | | | | | | | | | | |

| **Table A2. Routine, voluntary HIV screening in the French general population: base case analysis** | | | | | |
| --- | --- | --- | --- | --- | --- |
| **Variable** | | **“Current practice”** | **“Current practice” and screen once** | **“Current practice” and screen every 5 years** | **“Current practice” and screen annually** |
| **Mean CD4 count at diagnosis, cells/μl** | |  |  |  |  |
|  | All cases | 370 | 379 | 400 | 444 |
|  | Prevalent cases | 309 | 362 | 364 | 379 |
|  | Incident cases | 382 | 382 | 407 | 456 |
| **Mechanism of HIV diagnosis, % ^a^** | |  |  |  |  |
|  | Non-routine testing | 70 | 65 | 51 | 25 |
|  | AIDS-defining opportunistic disease | 25 | 23 | 17 | 9 |
|  | Screening program | 0 | 8 | 28 | 64 |
|  | Never diagnosed with HIV | 5 | 5 | 4 | 2 |
| ^a^ Numbers do not all add to 100%, due to rounding. | | | | | |

| **Table A3. Routine, voluntary HIV screening in the French general population and selected sub-populations: univariate sensitivity analyses ^a^** | | | | | | | |
| --- | --- | --- | --- | --- | --- | --- | --- |
|  |  |  | **Undiscounted** |  | **Discounted** | | |
| **Screening frequency** | |  | **Mean survival,**  **life-months** |  | **Mean quality-adjusted survival, QALM** | **Mean lifetime costs per person, 2007 €** | **Cost-effectiveness (€/QALY)** |
| *Incidence = 0.067/100PY (base case = 0.013/100PY)* | | | | | |  |  |
|  | Current practice |  | 475.78 |  | 267.86 | 4,590 | --- |
|  | Screen once |  | 475.80 |  | 267.87 | 4,640 | Dominated ^b^ |
|  | Screen every 5 years |  | 475.94 |  | 267.92 | 4,860 | 58,800 |
|  | Screen annually |  | 476.09 |  | 267.95 | 5,560 | 281,800 |
| *Incidence = 0.135/100PY (base case = 0.013/100PY)* | | | | | |  |  |
|  | Current practice |  | 471.72 |  | 266.77 | 8,650 | --- |
|  | Screen once |  | 471.74 |  | 266.78 | 8,700 | Dominated ^b^ |
|  | Screen every 5 years |  | 471.98 |  | 266.85 | 8,990 | 49,100 |
|  | Screen annually |  | 472.27 |  | 266.92 | 9,790 | 138,800 |
|  | | | | | |  |  |
| **Table A3. (continued)** | | | | | | | |
|  |  |  | **Undiscounted** |  | **Discounted** | | |
| **Screening frequency** | |  | **Mean survival, months** |  | **Mean quality-adjusted survival, QALM** | **Mean lifetime costs per person, 2007 €** | **Cost-effectiveness (€/QALY)** |
| *Undiagnosed HIV prevalence among MSM = 0.85% (base case = 1.70%)* | | | | | |  |  |
|  | Current practice |  | 393.92 |  | 242.36 | 54,930 | --- |
|  | Screen once |  | 393.91 |  | 242.38 | 55,050 | 80,100 |
|  | Screen every 5 years |  | 394.08 |  | 242.41 | 55,330 | 94,200 |
|  | Screen annually |  | 394.53 |  | 242.49 | 56,160 | 121,900 |
| *Undiagnosed HIV prevalence among IDU = 9.25% (base case = 6.17%)* | | | | | |  |  |
|  | Current practice |  | 443.55 |  | 254.76 | 36,760 | --- |
|  | Screen once |  | 445.05 |  | 255.74 | 39,380 | 32,100 |
|  | Screen every 5 years |  | 445.70 |  | 255.99 | 40,180 | 38,600 |
|  | Screen annually |  | 446.76 |  | 256.49 | 42,020 | 44,000 |
|  | | | | | |  |  |
|  | | | | | |  |  |
| **Table A3. (continued)** | | | | | | | |
|  |  |  | **Undiscounted** |  | **Discounted** | | |
| **Screening frequency** | |  | **Mean survival, months** |  | **Mean quality-adjusted survival, QALM** | **Mean lifetime costs per person, 2007 €** | **Cost-effectiveness (€/QALY)** |
| *Rate of HIV test acceptance = 20% (base case = 79%)* | | | | | |  |  |
|  | Current practice |  | 479.13 |  | 268.77 | 1,290 | --- |
|  | Screen once |  | 479.13 |  | 268.77 | 1,300 | 52,100 |
| *Rate of HIV test acceptance = 90% (base case = 79%)* | | | | | |  |  |
|  | Current practice |  | 479.13 |  | 268.77 | 1,290 | --- |
|  | Screen once |  | 479.14 |  | 268.78 | 1,350 | 65,200 |
| *Additional HIV screening program start-up cost = €3.50/person (20 million HIV tests)* | | | | | | |  |
|  | Current practice |  | 479.13 |  | 268.77 | 1,290 | --- |
|  | Screen once |  | 479.14 |  | 268.78 | 1,350 | 61,200 |
| *Additional HIV screening program start-up cost = €7.00/person (10 million HIV tests)* | | | | | | |  |
|  | Current practice |  | 479.13 |  | 268.77 | 1,290 | --- |
|  | Screen once |  | 479.14 |  | 268.78 | 1,350 | 64,900 |
| **Table A3. (continued)** | | | | | | | |
|  |  |  | **Undiscounted** |  | **Discounted** | | |
| **Screening frequency** | |  | **Mean survival, months** |  | **Mean quality-adjusted survival, QALM** | **Mean lifetime costs per person, 2007 €** | **Cost-effectiveness (€/QALY)** |
| *HIV test sensitivity = 97% (base case = 100%)* | | | | | |  |  |
|  | Current practice |  | 479.13 |  | 268.77 | 1,290 | --- |
|  | Screen once |  | 479.14 |  | 268.77 | 1,340 | 77,200 |
| *HIV test specificity = 95% (base case = 99%)* | | | | | |  |  |
|  | Current practice |  | 479.13 |  | 268.77 | 1,290 | --- |
|  | Screen once |  | 479.14 |  | 268.77 | 1,350 | 82,200 |
| *HIV test specificity = 100% (base case = 99%)* | | | | | |  |  |
|  | Current practice |  | 479.13 |  | 268.77 | 1,290 | --- |
|  | Screen once |  | 479.15 |  | 268.78 | 1,340 | 52,700 |
| *Secondary HIV transmission rates halved* | | | | | |  |  |
|  | Current practice |  | 479.45 |  | 268.80 | 1,130 | --- |
|  | Screen once |  | 479.47 |  | 268.81 | 1,190 | 59,200 |
| **Table A3. (continued)** | | | | | | | |
|  |  |  | **Undiscounted** |  | **Discounted** | | |
| **Screening frequency** | |  | **Mean survival, months** |  | **Mean quality-adjusted survival, QALM** | **Mean lifetime costs per person, 2007 €** | **Cost-effectiveness (€/QALY)** |
| *Secondary HIV transmission rates doubled* | | | | | |  |  |
|  | Current practice |  | 478.48 |  | 268.70 | 1,610 | --- |
|  | Screen once |  | 478.50 |  | 268.71 | 1,660 | 54,000 |
| *No secondary HIV transmission when HIV RNA levels are <500 copies/ml* | | | | | |  |  |
|  | Current practice |  | 479.15 |  | 268.77 | 1,280 | --- |
|  | Screen once |  | 479.17 |  | 268.78 | 1,330 | 56,900 |
| *Mean CD4 count in patients diagnosed with asymptomatic HIV infection = 380/µl (base case = 439/µl)* | | | | | | | |
|  | Current practice |  | 479.13 |  | 268.77 | 1,290 | --- |
|  | Screen once |  | 479.14 |  | 268.77 | 1,340 | 84,000 |
| *ART initiation at CD4 count <500/µl or severe opportunistic disease (base case = <350/µl or severe opportunistic disease)* | | | | | | | |
|  | Current practice |  | 479.15 |  | 268.77 | 1,310 | --- |
|  | Screen once |  | 479.16 |  | 268.78 | 1,370 | 77,700 |
| **Table A3. (continued)** | | | | | | | |
|  |  |  | **Undiscounted** |  | **Discounted** | | |
| **Screening frequency** | |  | **Mean survival, months** |  | **Mean quality-adjusted survival, QALM** | **Mean lifetime costs per person, 2007 €** | **Cost-effectiveness (€/QALY)** |
| *Lower bound of efficacy estimates for ART regimens 3-6 (60% / 50% / 20% / 10%)* | | | | | | | |
|  | Current practice |  | 479.09 |  | 268.76 | 1,280 | --- |
|  | Screen once |  | 479.10 |  | 268.77 | 1,330 | 79,800 |
| *Upper bound of efficacy estimates for ART regimens 3-6 (90% / 70% / 50% / 40%)* | | | | | | | |
|  | Current practice |  | 479.22 |  | 268.79 | 1,290 | --- |
|  | Screen once |  | 479.23 |  | 268.80 | 1,340 | 92,800 |
| QALM: quality-adjusted life-month; QALY: quality-adjusted life-year; PY: person-year  ^a^ All results incorporate the favorable effects of routine HIV screening on secondary HIV transmission. The cost-effectiveness results shown are not calculable, due to rounding.  ^b^ A dominated strategy leads to higher costs and equal or lower quality-adjusted survival than some combination of other strategies. | | | | | | | |

**Figure Legend**

**Figure A1: Conceptual framework for the Screening Module.** A random number generator assigns each incoming member of the population four, HIV-related event times. A series of “IF/THEN” statements reconciles the sequencing of these events and determines if and when HIV infection takes place, the time and frequency of all HIVCTR activities, and times of HIV screen detection and patient eligibility for therapy. For HIV-infected individuals, this information is conveyed to the Disease Module, which then simulates the progress of HIV disease, clinical care, and all associated costs. For all individuals, the Screening Module records cost and performance data on HIVCTR services.

**Figure A1.**
